# Zieve’s Syndrome in a Patient With Alcohol Use Disorder and Alcohol-Associated Cirrhosis: A Case Report and Review of the Literature

**DOI:** 10.7759/cureus.106971

**Published:** 2026-04-13

**Authors:** Noor Albusta

**Affiliations:** 1 Internal Medicine, Beth Israel Lahey Health, Burlington, USA

**Keywords:** alcohol use disorder, cirrhosis, hemolytic anemia, hyperbilirubinemia, transfusion-refractory anemia, zieve’s syndrome

## Abstract

Zieve’s syndrome is a rare and reversible cause of hemolytic anemia classically defined by the triad of hemolytic anemia, hyperbilirubinemia, and transient hyperlipidemia in the setting of recent heavy alcohol use. It is frequently underrecognized, particularly in patients with alcohol-associated liver cirrhosis, where anemia is often attributed to gastrointestinal bleeding, hypersplenism, or bone marrow dysfunction. This syndrome represents an important diagnostic pitfall because transfusion-refractory anemia may be misattributed to gastrointestinal bleeding or autoimmune hemolytic anemia, leading to unnecessary invasive procedures. We present a 48-year-old man with alcohol use disorder and decompensated alcohol-associated liver cirrhosis who presented with lethargy and severe, transfusion-refractory anemia. Despite multiple transfusions, his hemoglobin demonstrated minimal and transient improvement. An extensive evaluation, including esophagogastroduodenoscopy, colonoscopy, direct antiglobulin testing (DAT), and workup for inherited and acquired hemolytic disorders, excluded gastrointestinal bleeding, autoimmune hemolysis, and other causes of hemolysis. Laboratory findings demonstrated DAT-negative hemolytic anemia with marked hyperbilirubinemia and paradoxical hyperlipidemia, while bone marrow biopsy revealed erythroid hyperplasia without evidence of myelodysplasia. With supportive care and alcohol cessation, the patient’s hemoglobin improved without further transfusion requirements. This case highlights Zieve’s syndrome as an important diagnostic consideration in patients with alcohol-associated cirrhosis presenting with transfusion-refractory anemia, as early recognition can prevent unnecessary invasive procedures, and emphasizes alcohol cessation as the cornerstone of management.

## Introduction

Zieve’s syndrome is a rare but reversible cause of hemolytic anemia associated with alcohol use disorder. Originally described in 1958, it is classically characterized by the triad of hemolytic anemia, hyperbilirubinemia, and transient hyperlipidemia in the setting of recent heavy alcohol consumption [1-3]. Despite being recognized for decades, Zieve’s syndrome remains underdiagnosed in modern clinical practice [4]. Patients with underlying liver disease frequently present with anemia due to a variety of more common etiologies, including gastrointestinal bleeding, hypersplenism, nutritional deficiencies, and bone marrow suppression, often leading clinicians away from considering this diagnosis [5].

The syndrome represents a diagnostic pitfall because its presentation can closely mimic acute gastrointestinal hemorrhage or autoimmune hemolytic anemia, particularly in patients with portal hypertension and known varices [6]. Furthermore, Zieve’s syndrome must be distinguished from spur cell anemia, another hemolytic process seen in advanced cirrhosis, which is characterized by acanthocytes on peripheral smear and carries a poor prognosis, unlike the reversible hemolysis of Zieve’s syndrome [5]. As a result, patients may undergo repeated transfusions, invasive procedures, and extensive diagnostic testing despite the condition being self-limited with alcohol cessation [7]. Recognition of Zieve’s syndrome is critical, as early diagnosis can prevent unnecessary interventions and allow for appropriate management focused on supportive care and abstinence [8]. We present a case of severe, transfusion-refractory anemia in a patient with decompensated alcohol-associated cirrhosis, highlighting the diagnostic challenges and clinical features that underscore this underrecognized entity.

## Case presentation

A 48-year-old man with a history of alcohol use disorder and alcohol-associated cirrhosis complicated by ascites and known grade I non-bleeding esophageal varices presented with lethargy, weakness, and dizziness. He had a history of chronic anemia and a prior admission for anemia attributed to chronic disease. The patient reported a long-standing history of heavy alcohol use, consuming approximately one bottle of tequila daily (equivalent to approximately 280-300 grams of ethanol per day) up until the time of presentation. He denied melena, hematemesis, hematochezia, abdominal pain, or other symptoms suggestive of acute gastrointestinal bleeding. His home medications included lactulose, rifaximin, furosemide, and spironolactone; he was not taking any medications known to cause drug-induced hemolysis.

On presentation, he was hemodynamically stable and breathing comfortably on room air. Physical examination was notable for scleral icterus, pallor, and abdominal distension consistent with ascites, without signs of active bleeding. Laboratory evaluation revealed severe anemia with a hemoglobin level of 5.4 g/dL, a mean corpuscular volume of 89 fL, baseline thrombocytopenia with a platelet count of 78 × 10⁹/L, and a white blood cell count of 8 × 10⁹/L. He was found to have marked hyperbilirubinemia with a total bilirubin of 21.7 mg/dL, predominantly indirect (indirect bilirubin: 17.9 mg/dL), as well as an elevated international normalized ratio of 2.2. Additional hemolysis markers demonstrated an elevated lactate dehydrogenase (LDH) of 485 U/L (reference range: 140-280 U/L) and an undetectable haptoglobin of <10 mg/dL (reference range: 30-200 mg/dL). Vitamin B12 level was 512 pg/mL, and folate level was 9.8 ng/mL. Iron studies revealed an elevated serum iron of 186 µg/dL, reduced total iron-binding capacity of 180 µg/dL, elevated transferrin saturation of 72%, and markedly elevated ferritin of 1,120 ng/mL. An admission lipid panel demonstrated significant hyperlipidemia, with triglycerides of 620 mg/dL, total cholesterol of 320 mg/dL, low-density lipoprotein (LDL) cholesterol of 105 mg/dL, and high-density lipoprotein (HDL) cholesterol of 32 mg/dL (Table 1).

**Table 1:** Laboratory findings on admission Hb: hemoglobin, MCV: mean corpuscular volume, WBC: white blood cell count, INR: international normalized ratio, TIBC: total iron-binding capacity, LDL: low-density lipoprotein, HDL: high-density lipoprotein

Parameter	Value	Reference range
Hb	5.4 g/dL	13.5-17.5 g/dL
MCV	89 fL	80-100 fL
Platelet count	78 × 10⁹/L	150-400 × 10⁹/L
WBC	8 × 10⁹/L	4-11 × 10⁹/L
Total bilirubin	21.7 mg/dL	0.2-1.2 mg/dL
Indirect bilirubin	17.9 mg/dL	0.2-0.8 mg/dL
INR	2.2	0.8-1.2
Vitamin B12	512 pg/mL	200-900 pg/mL
Folate	9.8 ng/mL	3-17 ng/mL
Serum iron	186 µg/dL	60-170 µg/dL
TIBC	180 µg/dL	240-450 µg/dL
Transferrin saturation	72%	20%-50%
Ferritin	1,120 ng/mL	30-400 ng/mL
Triglycerides	620 mg/dL	<150 mg/dL
Total cholesterol	320 mg/dL	<200 mg/dL
LDL	105 mg/dL	<130 mg/dL
HDL	32 mg/dL	>40 mg/dL

The patient received multiple transfusions for symptomatic anemia, ultimately totaling five units of packed red blood cells. His hemoglobin increased modestly from 5.4 g/dL to 6.3 g/dL following transfusion, reflecting a limited and transient response. The expected hemoglobin increment is approximately 1 g/dL per unit of packed red blood cells transfused; thus, an increase of only 0.9 g/dL after five units represents a markedly inadequate response (observed increment of 0.9 g/dL versus expected increment of 5-7 g/dL), consistent with ongoing hemolysis rather than blood loss. Further evaluation revealed a reticulocyte count of 9.9% with a reticulocyte production index of 3.1. Direct antiglobulin testing (DAT), eluate testing, and microDAT were negative, arguing against autoimmune hemolytic anemia. Peripheral blood smear review demonstrated macrocytosis and occasional target cells, but no schistocytes or acanthocytes (spur cells).

**Figure 1:**
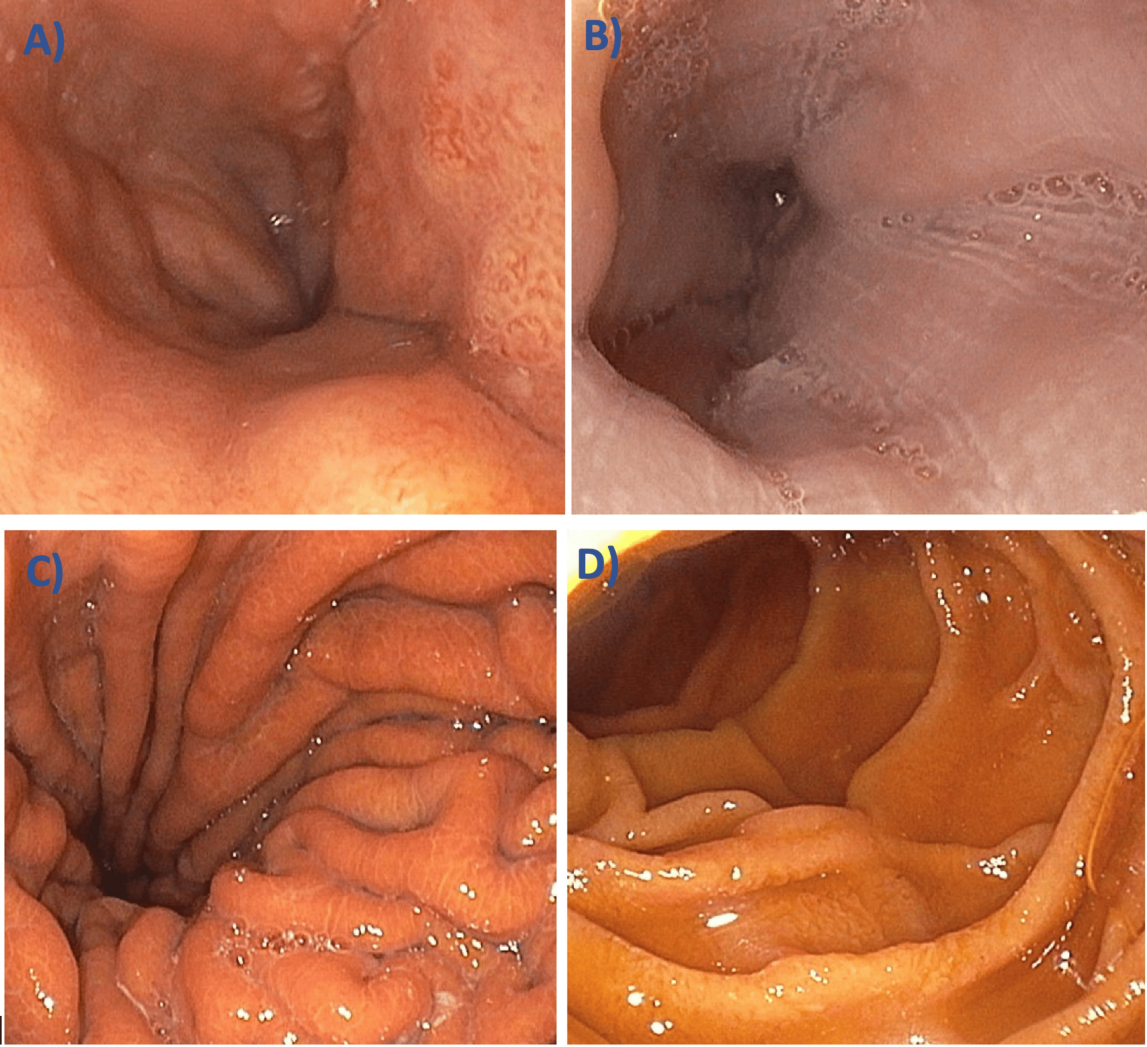
Upper endoscopy findings demonstrating (A) small grade I esophageal varices in the lower third of the esophagus, (B) grade I esophageal varices in the middle third of the esophagus, (C) gastric mucosa with mild portal hypertensive gastropathy without erosions or stigmata of bleeding, and (D) normal-appearing duodenal mucosa; no evidence of active or recent gastrointestinal bleeding was identified

An extensive hemolysis workup was performed and was negative for glucose-6-phosphate dehydrogenase deficiency, pyruvate kinase deficiency, paroxysmal nocturnal hemoglobinuria, and cryoglobulinemia. Given concern for occult gastrointestinal bleeding in the setting of cirrhosis, the patient underwent inpatient esophagogastroduodenoscopy, which demonstrated small grade I esophageal varices and mild portal hypertensive gastropathy without evidence of active or recent bleeding (Figure 1). Colonoscopy similarly revealed no bleeding source.

Because of the persistent anemia and lack of a clear etiology, a bone marrow biopsy was performed. This demonstrated a normocellular marrow with trilineage hematopoiesis and erythroid hyperplasia, without morphologic evidence of myelodysplastic neoplasm or infiltrative disease.

In the absence of an identifiable bleeding source, autoimmune hemolysis, inherited hemolytic disorders, or intrinsic marrow pathology, the patient’s anemia was attributed to non-autoimmune hemolytic anemia related to alcohol use, consistent with Zieve’s syndrome. The presence of the complete diagnostic triad (hemolytic anemia, hyperbilirubinemia, and hyperlipidemia) in the setting of recent heavy alcohol consumption supported this diagnosis. Management focused on supportive care and strict alcohol cessation, and no corticosteroids or immunosuppressive therapies were initiated.

At the time of discharge, his hemoglobin had improved to 7.1 g/dL without further transfusions, and his bilirubin levels were trending downward. At four-week outpatient follow-up, his hemoglobin had increased to 10.1 g/dL with sustained alcohol abstinence, and his total bilirubin had further improved to 7.6 mg/dL. Repeat lipid panel demonstrated improvement in lipid levels, with triglycerides of 150 mg/dL, total cholesterol of 180 mg/dL, LDL cholesterol of 110 mg/dL, and HDL cholesterol of 42 mg/dL. The patient continued to demonstrate clinical and hematologic improvement at outpatient hematology follow-up.

## Discussion

Zieve’s syndrome remains an underrecognized cause of hemolytic anemia, particularly in patients with alcohol use disorder and alcohol-associated liver cirrhosis. Although described more than six decades ago, it continues to represent a diagnostic pitfall due to overlapping clinical features with more common etiologies of anemia in this population [2,4]. Patients with cirrhosis frequently have multiple concurrent contributors to anemia, including portal hypertensive gastrointestinal bleeding, hypersplenism, nutritional deficiencies, and bone marrow suppression, which can obscure recognition of alcohol-related hemolysis [5]. The presence of portal hypertension, varices, and coagulopathy often biases clinicians toward gastrointestinal bleeding as the presumed cause, even in the absence of overt hemorrhage or objective endoscopic evidence of bleeding [6]. This diagnostic anchoring may lead to repeated transfusions and invasive procedures before alternative causes of anemia are adequately considered [7].

The pathophysiology of Zieve’s syndrome is incompletely understood and is believed to be multifactorial. Alcohol and its metabolite acetaldehyde can induce direct oxidative damage to erythrocyte membranes, increasing red blood cell fragility and promoting hemolysis [9]. Recent evidence demonstrates that ethanol directly induces hemolysis and primes red blood cells for erythrophagocytosis through eryptosis, marked by externalization of phosphatidylserine, creating a self-perpetuating cycle of red cell destruction [9]. In addition, alterations in lipid metabolism associated with heavy alcohol use result in abnormal incorporation of cholesterol and phospholipids into erythrocyte membranes, producing morphologic abnormalities that predispose red blood cells to premature destruction. Studies have demonstrated that erythrocyte membrane cholesterol-to-phospholipid ratios are increased in chronic alcoholics, with strong positive associations between membrane lipid peroxidation and hemolysis [10].

The transient hyperlipidemia observed in Zieve’s syndrome results from acute alcohol-induced disruption of hepatic lipid metabolism. It is associated with profound alterations in lipoprotein composition, including extreme decreases in plasma lecithin-cholesterol acyltransferase activity, resulting in reduced cholesteryl ester formation and accumulation of unesterified cholesterol. These changes produce abnormal triglyceride-rich lipoproteins that gradually return to baseline with clinical improvement [10]. The presence of hyperlipidemia in a cirrhotic patient is paradoxical and diagnostically important, as advanced cirrhosis typically causes hypolipidemia due to impaired hepatic synthetic function [5]. This contrast helps distinguish Zieve’s syndrome from complications of chronic liver disease alone and should prompt consideration of this diagnosis.

Clinically, patients with Zieve’s syndrome most often present with fatigue, weakness, jaundice, and severe anemia. Laboratory evaluation typically demonstrates DAT-negative hemolytic anemia with hyperbilirubinemia, transient hyperlipidemia, and a poor or transient response to transfusion [3,7]. A practical clinical clue is the observation of a hemoglobin increment well below the expected 1 g/dL per unit transfused; in this case, an increase of only 0.9 g/dL following five units of packed red blood cells strongly suggests ongoing hemolysis rather than blood loss [11]. Failure of hemoglobin levels to improve appropriately following transfusion should prompt consideration of ongoing hemolysis rather than blood loss as the primary driver of anemia. Continued transfusion may provide only temporary benefit while exposing patients to unnecessary risks, including volume overload and transfusion-related complications [8].

Improvement in hemoglobin, bilirubin, and lipid levels following alcohol cessation is a hallmark feature of Zieve’s syndrome and serves as both a diagnostic clue and a therapeutic endpoint. Studies have demonstrated that serum hemolysis diminishes after approximately one week of alcohol withdrawal, with parallel declines in markers of macrophage-mediated erythrophagocytosis [9]. Serial lipid measurements serve both diagnostic and monitoring functions, as resolution of hyperlipidemia parallels improvement in hemolysis and validates the diagnosis retrospectively. Recognition of this clinical trajectory is critical, as management is primarily supportive and centered on strict abstinence from alcohol. Unlike autoimmune hemolytic anemia, corticosteroids and other immunosuppressive therapies have no established role in the treatment of Zieve’s syndrome and may expose patients to avoidable harm [7]. Early identification can therefore prevent inappropriate therapies and reduce unnecessary diagnostic procedures such as repeated endoscopic evaluations.

It is important to distinguish Zieve’s syndrome from spur cell anemia, another hemolytic process associated with advanced liver disease. Spur cell anemia is characterized by the presence of acanthocytes (spur cells) on peripheral smear and carries a poor prognosis, whereas Zieve’s syndrome is typically reversible with alcohol cessation. The absence of acanthocytes on peripheral smear in this case, along with the presence of hyperlipidemia and clinical improvement following abstinence, supported the diagnosis of Zieve’s syndrome rather than spur cell anemia [5].

To date, most reported cases of Zieve’s syndrome have occurred in patients with alcoholic hepatitis or steatohepatitis without established cirrhosis. This case contributes to the literature by demonstrating that the syndrome can occur in the setting of decompensated alcohol-associated cirrhosis, where the differential diagnosis for anemia is considerably broader and diagnostic delay is more likely [1,9].

The findings in this case are consistent with previously reported features of Zieve’s syndrome, including DAT-negative hemolytic anemia, marked hyperbilirubinemia, and transient hyperlipidemia in the setting of heavy alcohol use. Similar to prior reports, this patient demonstrated a poor response to transfusion, supporting ongoing hemolysis rather than blood loss as the primary driver of anemia. This case further expands the literature by highlighting the diagnostic challenge in patients with advanced cirrhosis, where competing etiologies of anemia may delay recognition.

## Conclusions

This case underscores the importance of maintaining a high index of suspicion for Zieve’s syndrome in patients with alcohol use disorder who present with severe, transfusion-refractory anemia, hyperbilirubinemia, and paradoxical hyperlipidemia. Documentation of the complete diagnostic triad (hemolytic anemia, jaundice, and transient hyperlipidemia) strengthens diagnostic confidence and facilitates the timely diagnosis of Zieve’s syndrome. Prompt recognition may reduce unnecessary interventions, shorten hospital stays, and reinforce alcohol cessation as the definitive treatment.
